# Improvement in quality of life and cardiac function after catheter ablation for asymptomatic persistent atrial fibrillation

**DOI:** 10.1002/joa3.12457

**Published:** 2020-12-11

**Authors:** Naoaki Onishi, Shokan Kyo, Maki Oi, Toshikazu Jinnai, Maiko Kuroda, Yukiko Shimizu, Sari Imamura, Takeshi Harita, Suguru Nishiuchi, Koji Hanazawa, Toshihiro Tamura, Chisato Izumi, Yoshihisa Nakagawa, Kazuaki Kaitani

**Affiliations:** ^1^ Division of Cardiology Tenri Hospital Tenri Japan; ^2^ Japanese Red Cross Otsu Hospital Otsu Japan; ^3^ Hyogo Prefectural Amagasaki General Medical Center Amagasaki Japan; ^4^ Hidaka Hospital Gobo Japan; ^5^ Kitano Hospital Osaka Japan; ^6^ Japanese Red Cross Wakayama Medical Center Wakayama Japan; ^7^ National Cerebral and Cardiovascular Center Suita Japan; ^8^ Shiga University of Medical Science Otsu Japan

**Keywords:** atrial fibrillation, B‐type natriuretic peptide, catheter ablation, quality of life, reverse remodeling

## Abstract

**Background:**

Catheter ablation (CA) for atrial fibrillation (AF) is widely performed. However, the indication for CA in patients with asymptomatic persistent AF is still controversial.

**Methods:**

Among 259 consecutive patients who were hospitalized for initial CA of AF, a total of 45 patients who had asymptomatic persistent AF were retrospectively analyzed. Quality of life (QOL) before and 1 year after CA was evaluated, and changes in the cardiac function over 5 years after CA were also examined. QOL was assessed using the AF QOL questionnaire (AFQLQ) developed by the Japanese Heart Rhythm Society. In addition, cardiac function was assessed by measuring the plasma B‐type natriuretic peptide (BNP) level, left ventricular ejection fraction (LVEF), left atrial diameter (LAD) with transthoracic echocardiogram, and left atrial (LA) volume with computed tomography (CT).

**Results:**

The AFQLQ significantly improved after CA in terms of “symptom frequency” and “activity limits and mental anxiety.” The plasma BNP level, LVEF, and LAD significantly improved in the first 3 months after the first CA, with no significant changes thereafter (from 149.0 pg/dL [95% confidence intervals {CI}, 114.5‐183.5 pg/dL] to 49.8 pg/dL [95% CI, 26.5‐70.1], *P* < .0001; from 60.8% [95% CI, 58.1%–63.6%] to 65.0% [95% CI, 62.6‐67.4], *P* = .001; and from 41.3 mm [95% CI, 39.7‐42.9] to 36.8 [95% CI, 34.5‐39.1 mm], *P* < .0001, respectively). LA volume revealed LA reverse remodeling after CA.

**Conclusion:**

Improvement in the QOL and cardiac function after CA of asymptomatic persistent AF was revealed. Asymptomatic persistent AF should be appropriately treated by CA.

## Graphical abstract

Before and after catheter ablation (CA) for asymptomatic persistent atrial fibrillation, quality of life (QOL) was assessed using the AF QOL questionnaire (AFQLQ), and cardiac function was assessed by measuring the plasma B‐type natriuretic peptide (BNP) level, left ventricular ejection fraction (LVEF), and left atrial diameter (LAD) with transthoracic echocardiogram and left atrial (LA) volume with computed tomography (CT). Improvement in QOL and cardiac function after CA for asymptomatic persistent AF was revealed.

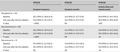

## 1 INTRODUCTION

Catheter ablation (CA) for atrial fibrillation (AF) is the first‐line therapy, especially for symptomatic AF refractory or intolerant to at least one class I or III antiarrhythmic drug (AAD).^1^ Moreover, quality of life (QOL) has been reported to improve after CA for symptomatic paroxysmal AF (PAF).^2^ However, the improvement of QOL and long‐term prognosis after CA of asymptomatic persistent AF remains unknown. In addition, sinus rhythm (SR) maintenance rate after CA in persistent AF was lower than in PAF.^3^ Therefore, in clinical practice, the indication for CA in asymptomatic persistent AF patients is often controversial. This study aimed to examine whether CA improves QOL and cardiac function in patients with asymptomatic persistent AF.

## 2 METHODS

### 2.1 Study design and population

This was a retrospective single‐center study. A total of 259 consecutive patients underwent an initial radiofrequency (RF) CA of AF in Tenri Hospital from January 2012 to March 2014; 165 patients had PAF. Of the remaining 94 persistent AF patients, 45 had asymptomatic AF. This study was conducted on the 45 patients. Consent for the treatment was obtained after explaining the risks of the ablation, including that it was an invasive treatment and that the outcome of non‐PAF has a higher recurrence than that of PAF, as well as the benefit of a sequential discontinuation of the anticoagulation according to the CHADS_2_ score.^4^

### 2.2 The definition of asymptomatic persistent AF

Paroxysmal AF was defined as that which terminated spontaneously or under AADs within 7 days of onset. Persistent AF was defined as that lasting for >7 days; in particular, long‐standing persistent AF was defined as that lasting >1 year.^5^ Asymptomatic persistent AF was referred to as persistent AF when incidentally discovered during routine clinical examinations or detected by screening and recorded for ≥30 seconds via the electrocardiogram (ECG).^6^

### 2.3 Quality of life measurement

Quality of life was assessed before and 1 year after the first CA using an AF‐specific QOL assessment method, that is, AF QOL questionnaire (AFQLQ, developed by the Japanese Society of Electrocardiology, currently Japanese Heart Rhythm Society).^7^, ^8^, ^9^ The AFQLQ has three categories: AFQLQ1 evaluates symptom frequency (out of 24 scores), AFQLQ2 evaluates symptom severity (out of 18 scores), and AFQLQ3 evaluates activity limits and mental anxiety (out of 56 scores). Thus, the higher the score is, the better the QOL.

### 2.4 Assessment of cardiac function

The plasma B‐type natriuretic peptide (BNP) level, which reflects left ventricular end‐diastolic pressure and is known as a marker of heart failure, was examined.^10^, ^11^ In addition, left atrial (LA) volume was measured by contrast‐enhanced cardiac computed tomography (CT) scan as an indicator of LA remodeling.^12^ Moreover, left ventricular ejection fraction (LVEF) and LA diameter (LAD), which are indicators of the left ventricular systolic and diastolic function, respectively, were measured with transthoracic echocardiography (TTE).^13^ For the LVEF and LAD measurements using TTE, especially in the case of AF rhythm, we used measurements at the three consecutive beats with the least variability in the heart rate. LVEF was assessed by the modified Simpson's method, and LAD with the long‐axis view was measured at the end systole. Remeasurements of those parameters were conducted by experienced sonographers at our institution to certify the accuracy of the echocardiographic parameters.

### 2.5 Screening for sleep‐disordered breathing

Sleep‐disordered breathing (SDB) is known to be common in patients with AF.^14^ Therefore, SDB was screened as part of the information for the baseline patient characteristics by measuring the 3% oxygen desaturation index (ODI) using a high‐resolution pulse oximeter (PULSOX‐Me300; Minolta Co.), the night before AF ablation in our hospital.^15^

### 2.6 Ablation procedure

All patients had been on anticoagulation therapy (ACT) with warfarin or direct oral anticoagulants (DOACs) for at least 1 month before the ablation procedure. For warfarin, the prothrombin time‐international normalized ratio was adjusted to 2.0‐3.0. Before CA, the absence of any LA thrombi was confirmed by transesophageal echocardiography. If the patient was receiving warfarin, CA was performed without interruption of the anticoagulant. For the patients receiving a DOAC, the anticoagulant was interrupted only on the day of the CA.

A duo‐decapolar electrode catheter (IBI‐86010, St. Jude Medical, Inc, or BeeAT^TM^, Japan Lifeline Co., Ltd) was inserted through a 7‐F sheath from the right subclavian or internal jugular vein and positioned in the coronary sinus. An 8.5‐F Swartz^TM^ SL0^TM^ introducer sheath and Agilis^TM^ NxT steerable introducer (St. Jude Medical Inc) were inserted through the right femoral vein. For the blood pressure measurements, a 3‐F sheath was inserted through the right femoral artery. The BRK^TM^ needle (St. Jude Medical, Inc) or NRG^TM^ transseptal needle (Baylis Medical Company, Inc) was used for the fluoroscopy‐guided transseptal puncture. A 10 IU/kg body weight dose of heparin was administered before the transseptal puncture. To maintain the activated clotting time at 300‐350 seconds, heparinized saline was continuously infused during the procedure, and it was measured every 30 minutes to fine‐tune the heparin dose. CA was performed under sedation with dexmedetomidine infusion and respiratory control with adaptive support ventilation. During CA, intravenous isoproterenol was administered continuously (20‐25 μg/h), and fentanyl and thiopental were used as an analgesic and sedative, as appropriate, respectively, in addition to continuous intravenous dexmedetomidine. The pulmonary vein (PV) electrograms were monitored using a size‐adjustable decapolar circumferential mapping catheter (Lasso, Biosense Webster, Inc, or EPstar Libero, Japan Lifeline Co., Ltd). LA contrast imaging was performed, and CA was performed under the guidance of a three‐dimensional mapping system (CARTO XP or CARTO 3, Biosense Webster, Inc). Cardioversion was performed before PV isolation. PV isolation was performed during AF if SR recovery was unsuccessful. In such cases, SR was recovered by performing cardioversion during or after PV isolation. A 3.5‐mm externally irrigated‐tip ablation catheter (NAVISTAR Thermocool, Biosense Webster, Inc) was used to perform circumferential PV isolation. RF energy was delivered at 25‐35 W and a flow rate of 17‐30 mL/min with a maximal temperature of 40°C. The RF energy deliveries were limited to 20‐25 W on the LA posterior wall in close proximity to the esophageal temperature probe (SensiTherm, St. Jude Medical, Inc) and terminated when the esophageal temperature readings reached 39°C. Bidirectional conduction block between the LA and PV was confirmed using the pacing technique. A cavotricuspid isthmus ablation, superior vena cava isolation, LA linear ablation (Box isolation^16^), and mitral‐valve isthmus ablation were performed, per the operators’ discretion. After the completion of CA, protamine (50 mg) was administered to reverse the effect of heparin and all sheaths were removed.

### 2.7 Recurrent atrial tachyarrhythmias after CA

Recurrent atrial tachyarrhythmias were defined as that lasting for 30 seconds or requiring a repeat ablation, hospital admission, or unscheduled usage of Vaughan Williams class I or III AADs, excluding the events during the blanking period of 90 days after ablation. A repeat ablation during the blanking period of 90 days was discouraged.

### 2.8 Follow‐up

The patients were scheduled to receive periodic regular follow‐ups at the outpatient clinic of the centers, in which the first follow‐ups were performed at 1, 3, and 6 months and 1, 2, 3, 4, and 5 years after CA. A 12‐lead ECG was obtained at every visit. One‐channel ECGs were recorded for 2 weeks, twice daily, when the patients had symptoms suggestive of arrhythmias in the hospital, at hospital discharge, and at 3 months, 6 months, and 1 year using an ambulatory electrogram recorder (HCG‐801, OMRON Healthcare Co., Ltd). Per the outpatient physician's decision, 24‐hour Holter monitoring was performed at 6 months and 1 year and at 2, 3, 4, and 5 years. Blood sampling, including the plasma BNP level, was examined in the hospital at 3 and 6 months and at 1, 2, 3, 4, and 5 years. TTE was performed before CA and at 3 and 6 months and 1, 2‐3, and 4‐5 years after CA, and LVEF and LAD were measured. A contrast‐enhanced cardiac CT scan was conducted before and 6 months after CA to measure the LA volume. When patients were unable to visit the outpatient clinic, the follow‐up data were obtained by contacting the physicians in charge or the patients. The mean duration of ACT in the patients who discontinued ACT after CA was also investigated. In addition, the adverse events (all‐cause death, cardiovascular death, strokes, and heart failure hospitalization) during the follow‐up for all patients after CA were examined.

### 2.9 Statistical analysis

The data are presented as values and percentages, mean ± SD, or median with interquartile range (IQR). The Chi‐square or Fisher's exact test and Student's *t* test or Wilcoxon rank‐sum test were used to compare categorical and continuous variables, respectively, on the basis of their distribution. The Kaplan‐Meier method was used to estimate the recurrent atrial tachyarrhythmia event‐free rate. The paired t‐test was used to compare AFQLQ, plasma BNP level, CT LA volume, LVEF, and LAD before and after CA. They were described as mean and 95% CI for between‐group comparisons. Patients who died during the follow‐up were censored on the date of death or last contact. JMP 13.0 software (SAS Institute Inc) was used for all analyses. All statistical analyses were two‐tailed, and *P* < .05 was considered statistically significant.

## 3 RESULTS

### 3.1 Baseline characteristics

The baseline characteristics before CA are presented in Table 1. The mean age was 62.9 ± 8.6 years, and five patients (11.1%) were female. The median AF duration was 8.2 months (IQR, 3.0‐15.2), and 13 patients (28.9%) had long‐standing AF. The mean CHADS_2_ score was 1.0 ± 0.9. The plasma BNP level, LVEF, LAD, and CT LA volume were 149.0 ± 114.9 pg/dL, 60.8 ± 9.2%, 41.3 ± 5.3 mm, and 109.8 ± 31.6 mL, respectively. The median 3% ODI was 9.2 (IQR, 5.9‐13.3). In 36 patients (80.0%), the 3% ODI was ≥5.0 and the possibility of at least mild SDB was suggested.^17^

**TABLE 1:** Baseline characteristics

	n = 45
Age (y)	62.9 ± 8.6
Duration of AF (mo)	8.2 (3.0‐15.2)
Long‐standing persistent AF (%)	13 (28.9)
Female (%)	5 (11.1)
Heart failure (%)	5 (11.1)
Hypertension (%)	22 (48.9)
Diabetes (%)	6 (13.3)
Ischemic stroke (%)	5 (11.1)
Vascular disease (%)	2 (4.4)
CHADS_2_ score	1.0 ± 0.9
Dialysis	0 (0)
LVEF (%)	60.8 ± 9.2
LAD (mm)	41.3 ± 5.3
CT LA volume (mL)	109.8 ± 31.6
Hb (mg/dL)	14.8 ± 1.3
HbA1c (%)	5.8 ± 0.4
BNP (pg/dL)	149.0 ± 114.9
Creatinine (mg/dL)	0.89 ± 0.18
3% ODI	9.2 [5.9‐13.3]

Abbreviations: AF, atrial fibrillation; BNP, B‐type natriuretic peptide; CT, computed tomography; Hb, hemoglobin; LA, left atrium; LAD, left atrium diameter; LVEF, left ventricular ejection fraction; ODI, oxygen desaturation index.

### 3.2 Ablation procedural data

Ablation strategies used in the first CA are presented in Table 2. PV isolation was performed in all patients. Box isolation was performed in 11 patients (24.4%), and mitral‐valve isthmus ablation was performed in three patients (6.7%). In all 45 sessions in this study, there were no major complications, such as cardiac tamponade, acute thromboembolic events, or adverse effects of AADs.

**TABLE 2:** Ablation strategy

Ablation strategy	n (%)
PV isolation	45 (100)
Box isolation	11 (24.4)
SVC isolation	30 (66.7)
MVI block line	3 (6.7)
CTI block line	18 (40.0)

Abbreviations: CTI, cavotricuspid isthmus; MVI, mitral‐valve isthmus; PV, pulmonary vein; SVC, superior vena cava.

### 3.3 Follow‐up data

The median follow‐up period after the first ablation was 5.2 years (IQR, 4.9‐6.1), and the follow‐up rate was 100%.

### 3.4 Atrial tachyarrhythmia recurrence after the first and last sessions

**FIGURE 1:**
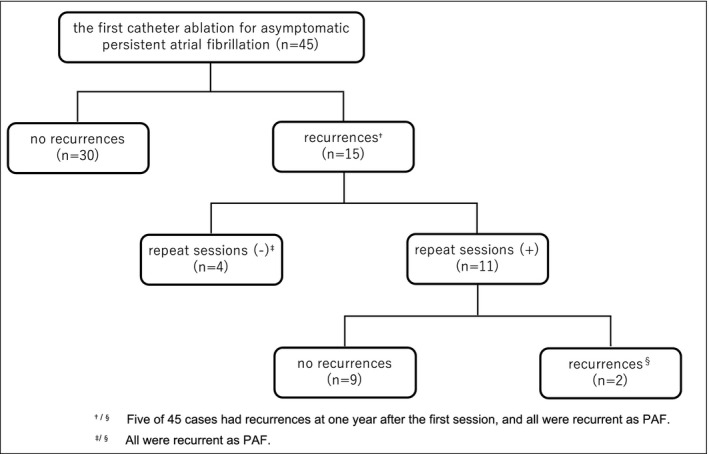
The flowchart of the recurrences of atrial tachyarrhythmias and repeat sessions after the first session. PAF, paroxysmal atrial fibrillation

**FIGURE 2:**
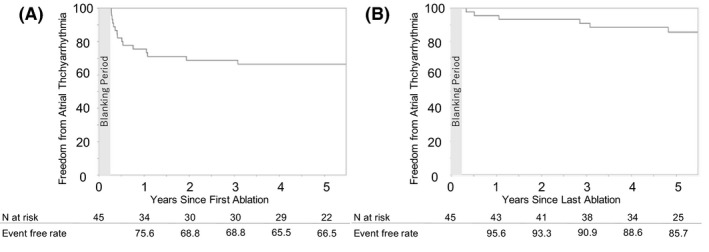
Freedom from atrial tachyarrhythmias (A, first session; B, last session)

The flowchart of the recurrences of atrial tachyarrhythmias and repeat sessions after the first CA is presented in Figure 1. Atrial tachyarrhythmia recurrences after the first CA were observed in 15 of 45 cases (33.3%). In 11 of those 15 cases (73.3%), a second session was performed, and more than second session was not performed. The number of mean sessions was 1.2 ± 0.2. At 1 year after the first CA, 5 of 45 cases (11.1%) had recurrences, and some of the cases included a second session within 1 year of the first CA. All of the five cases had recurrences as PAF, not persistent atrial tachyarrhythmias. Moreover, all six cases had recurrences after the last session (13.3%; four patients had recurrences after the first CA but did not receive a second session; two patients had recurrences after a second session), and their style of recurrences was also PAF. Atrial tachyarrhythmia‐free rate after the first and last sessions at 5 years was 66.5% and 85.7%, respectively (Figure 2A,B).

### 3.5 AFQLQ

AFQLQ1 (symptom frequency) and AFQLQ3 (activity limits and mental anxiety) significantly improved without any remarkable change in AFQLQ2 (symptom severity) in all patients. Similar results were obtained in 40 patients whose SR was maintained at 1 year after the first session, including cases with a second session within 1 year after the first session. In the remaining five patients who had recurrences, only AFQLQ3 improved, but there was no remarkable change in AFQLQ1 and AFQLQ2 (Table 3).

**TABLE 3:** Changes in QOL before and 1 y after first CA

	AFQLQ1	AFQLQ2	AFQLQ3
	Symptom frequency	Symptom severity	Activity limits and mental anxiety
All patients (n = 45)			
Baseline	20.1 (95% CI, 18.8‐21.4)	16.3 (95% CI, 15.7‐17.0)	46.8 (95% CI, 44.5‐49.1)
One year after the first ablation	23.4 (95% CI, 23.1‐23.7)	16.4 (95% CI, 15.9‐17.0)	50.9 (95% CI, 49.0‐52.8)
*P* value	<.0001	NS	.002
No recurrences (n = 40)			
Baseline	20.2 (95% CI, 18.9‐21.5)	16.4 (95% CI, 15.7‐17.1)	46.8 (95% CI, 44.2‐19.3)
One year after the first ablation	23.5 (95% CI, 23.2‐23.8)	16.5 (95% CI, 15.9‐17.1)	50.5 (95% CI, 48.4‐52.6)
*P* value	<.0001	NS	.01
Recurrences (n = 5)			
Baseline	19.4 (95% CI, 12.3‐26.5)	15.8 (95% CI, 13.4‐18.2)	47.0 (95% CI, 40.4‐53.6)
One year after the first ablation	22.6 (95% CI, 20.5‐24.7)	15.6 (95% CI, 13.7‐17.5)	54.0 (95% CI, 52.4‐55.5)
*P* value	NS	NS	.04

Abbreviations: CA, Catheter ablation; CI, confidence intervals; NS, not significant, ODI, oxygen desaturation index.

### 3.6 Plasma BNP level, CT LA volume, and echo parameters (LAD and LVEF)

**FIGURE 3:**
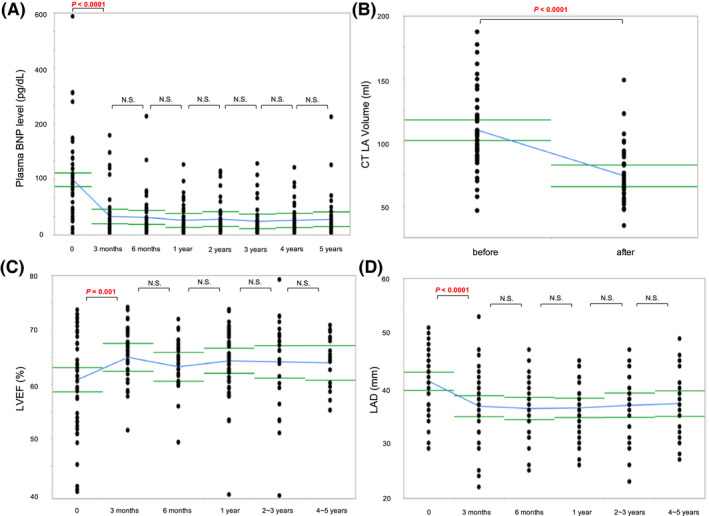
Changes in plasma BNP level (A), CT LA volume (B), LVEF (C), and LAD (D) before and after the first CA. The blue line represents the change in the mean value, and the green line represents the 95% confidence interval of the mean value. BNP, B‐type natriuretic peptide; CT, computed tomography; LA, left atrium; LAD, left atrium diameter; LVEF, left ventricular ejection fraction; NS, not significant

The plasma BNP level significantly decreased during the first 3 months after first CA (from 149.0 pg/dL [95% CI, 114.5‐183.5 pg/dL] to 49.8 pg/dL [95% CI, 26.5‐70.1 pg/dL], *P* < .0001), and thereafter, there was no significant difference during the 5 years (Figure 3A). The CT LA volume after CA became much smaller than before CA (from 109.8 mL [95% CI, 100.3‐119.3 mL] to 73.7 mL [95% CI, 66.7‐80.8 mL]; *P* < .0001; Figure 3B). Moreover, LVEF and LAD improved during the first 3 months after the first CA (from 60.8% [95% CI, 58.1‐63.6] to 65.0% [95% CI, 62.6‐67.4], *P* = .001; from 41.3 mm [95% CI, 39.7‐42.9 mm] to 36.8 mm [95% CI, 34.5‐38.1 mm], *P* < .0001, respectively), and thereafter, there was no significant difference during the 5 years (Figure 3C,D).

### 3.7 Discontinuation of ACT

In 28 patients (62.2%), the ACT was discontinued after the last ablation and the total anticoagulant time from the first CA was 344 ± 259 days in the 28 cases. Those patients’ CHADS_2_ score at baseline was significantly lower than the CHADS_2_ score of the remaining 17 patients (0.68 ± 0.72 vs 1.6 ± 0.8, *P* = .0004).

### 3.8 Adverse events during the follow‐up

There were two all‐cause deaths (4.4%; one asphyxia caused by aspiration and one lung cancer), but there were no cardiovascular deaths. The patients without a history of heart failure before CA were not hospitalized for heart failure after CA. Five patients (11.1%) had a history of heart failure or LVEF ≤ 40%^18^ before CA, and had no subjective symptoms such as shortness of breath or palpitations. Four of them (80.0%), who were maintained in SR after the last ablation, had no exacerbations of heart failure. One patient (20.0%) was hospitalized again because of heart failure after an AF recurrence. Five patients (11.1%) had a history of a cerebral infarction before CA but had no recurrence of the cerebral infarction after CA. For those five patients, ACT was continued even though SR was maintained. Lacunar infarction occurred in another patient (11.1%) who stopped ACT because of maintaining SR after the first CA. There were no cardiogenic cerebral infarctions or hemorrhagic events in any of the patients after CA during the follow‐up.

## 4 DISCUSSION

### 4.1 Main findings

In this study, AFQLQ in the asymptomatic persistent AF patients significantly improved after CA in terms of “symptom frequency” and “activity limits and mental anxiety”. Moreover, the plasma BNP level and LVEF improved, and LA volume reduction was observed.

### 4.2 QOL and AF

Several previous reports have indicated a significant improvement in QOL after CA in patients with AF using the 36‐item short‐form questionnaire (SF‐36).^2^, ^19^, ^20^, ^21^ SF‐36 is the most popular QOL assessment instrument in the world to measure the health status and is an excellent comprehensive measure of overall health.^22^ However, it is not necessarily suitable for a specific assessment of disease‐specific QOL changes associated with a given disease.^23^ AFQLQ is an AF‐specific QOL assessment method developed by the Japanese Society of Electrocardiology, currently Japanese Heart Rhythm Society, which has been reported to be better correlated with the ablation outcomes than a general QOL assessment such as SF‐36.^24^, ^25^

Miyazaki et al^24^ and Yagishita et al^25^ evaluated QOL after CA of persistent AF using the AFQLQ and reported that all three subsets of the AFQLQ significantly improved in the patients without recurrences but that there were no differences in those with recurrences. In contrast, in our study, symptom frequency (AFQLQ1) significantly improved when SR was maintained; however, symptom severity (AFQLQ2) did not change regardless of the maintenance of SR. In our study, asymptomatic persistent AF was defined as incidentally discovered persistent AF in accordance with the previous report,^6^ and all the patients reported no complaints during the medical interviews. The definition was similar in the paper by Yagishita et al^25^, who evaluated QOL after CA in asymptomatic persistent AF, and the present study. It is important to note that, even though AF was diagnosed by chance in those who denied having any complains in the medical interview, it did not mean that there were no symptoms of AF at all, because symptoms might be minimal and gradually be tolerated by self‐control. It is also important to note that only when SR is maintained may one realize that these symptoms were associated with AF.

Moreover, in this study, activity limits and mental anxiety (AFQLQ3) significantly improved after CA with or without recurrent atrial tachyarrhythmias. Miyazaki et al^24^ and Yagishita et al^25^ did not state whether the AF recurrence was persistent or paroxysmal in their articles. In our study, all patients with recurrences at 1 year after the first CA were paroxysmal. The recurrence pattern from a persistent to paroxysmal form sometimes leads to worsening AF symptoms. However, the AFQLQ among those recurrent patients did not become worse because the recurrence rate was limited. Therefore, the procedural success rate highly affected our conclusion. Our findings might suggest that our success rate or the indications were within a reasonable range for CA of asymptomatic AF.

A significant inverse association has been reported between physical activity and AF burden.^26^ Improvement in exercise performance is also known to promote serotonin secretion in the brain, leading to mental stability.^27^ Although we did not evaluate exercise performance using a treadmill test in this study, it is possible that the reduction in AF burden after CA may have led to an improvement in the activity and ultimately relief of the mental stability.

### 4.3 The improvement in cardiac function after CA

The plasma BNP level, LVEF, and LAD were found to improve early after CA and then be maintained in our study. Similar results were reported in the CAMTAF AF trial,^28^ wherein the plasma BNP level and LVEF improved in the first 1 month after the first CA for persistent AF patients whose LVEF was under 50%. In this study, most patients had a normal LVEF (60.8 ± 9.2%) and only five patients (11.1%) out of 45 had an LVEF under 50%. Even in the 40 patients with LVEF at ≥50%, there was a significant improvement in the plasma BNP level, LVEF, and LAD at 3 months after CA (from 140.5 pg/dL [95% CI, 104.8‐176.2 pg/dL] to 46.9 pg/dL [95% CI, 26.7‐67.2], *P* < .0001; from 63.0% [95% CI, 60.8‐65.3] to 66.4% [95% CI, 64.8‐68.0], *P* = .003; and from 41.0 mm [95% CI, 39.2‐42.7] to 36.9 [95% CI, 34.4‐39.4], *P* = .0002, respectively), and a significant reduction in CT volume after CA (from 108.8 mL [95% CI, 98.4‐119.2] to 75.9 mL [95% CI, 68.5‐83.4], *P* < .0001). We do not have the TTE data at 1 month after CA; hence, changes in those parameters at 1 month after CA were unknown in our study. However, we found a significant improvement in these parameters early after CA, even in patients with persistent AF and a relatively normal LVEF.

The reduction in the end‐systolic volume because of LVEF improvement suggested an acute effect of SR restoration and the possibility of left ventricular reverse remodeling.^28^ In addition, the decrease in LAD in TTE and the reduction in the CT LA volume after CA represent LA reverse remodeling.^12^ These series of improvements in cardiac function may predict a reduction in long‐term mortality and heart failure events.^29^, ^30^, ^31^

### 4.4 AF recurrence as PAF and decreasing AF burden

**FIGURE 4:**
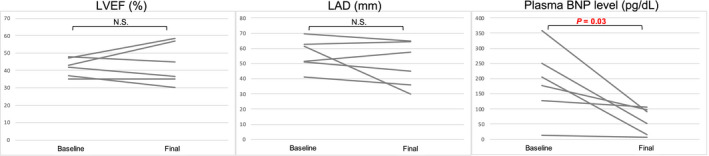
Changes in plasma BNP level, CT LA volume, LVEF, and LAD between baseline and final data in six patients who had recurrences after last ablation. BNP, B‐type natriuretic peptide; LAD, left atrium diameter; LVEF, left ventricular ejection fraction; NS, not significant

There were six patients who still had recurrences after the last session, and all recurrent arrhythmias were PAF, not persistent atrial tachyarrhythmias. The plasma BNP level, LVEF, and LAD between baseline and the final data were compared, and it revealed that LVEF and LAD did not improve but that the plasma BNP level improved (from 56.3% [95% CI, 45.5‐67.1] to 49.7% [95% CI, 34.0‐65.3], *P* = .27; from 42.0 [95% CI, 36.5‐47.5 mm] to 43.7 mm [95% CI, 31.2‐56.2], *P* = .66; and from 188.8 pg/dL [95% CI, 66.4‐311.2] to 61.6 pg/dL [95% CI, 16.5‐106.7], *P* = .03, respectively; Figure 4).

The plasma BNP level may be lower because CA makes the patients more health‐oriented, which may result in better medication adherence, improved hypertension owing to salt suppression, and appropriate use of continuous positive airway pressure in SDB patients, leading to improvements in heart failure. Furthermore, a reduction in AF burden has been reported to be associated with a decrease in the plasma BNP level.^32^ In this study, a reduction in AF burden owing to the change from persistent AF to PAF after CA may also be responsible for the improvement in the plasma BNP level. We can also expect to see an improvement in exercise performance and QOL in terms of “activity limits and mental anxiety” by a reduction in AF burden. The continuation of the ACT is essential for these patients who have recurrences as PAF even after multiple ablation sessions for persistent AF, and outpatient follow‐up is necessary. Long‐term outpatient follow‐up and management of asymptomatic persistent AF cases might often be a tough task. However, in our data, the change in the QOL scores correlated with the objective data such as the cardiac biomarkers and echocardiographic data. Therefore, the serial evaluation of the QOL score within 1 year after the treatment is important to ensure a safer longer follow‐up after CA of asymptomatic AF.

## 5 LIMITATIONS

This study had several limitations. First, the sample size was small. Second, detecting AF recurrence after CA in asymptomatic AF is difficult. Therefore, the recurrence rate might have been underestimated. Third, in this study, most patients (32 cases, 71.1%) had AF duration of <1 year. In addition, the mean CHADS_2_ score was 1.0 ± 0.9 with a low‐risk profile. We must be cautious when applying the results of this study to asymptomatic AF patients with longer AF duration or with higher CHADS_2_ scores. An interpretation of these patient groups requires further research. Fourth, the median AF duration might have been underestimated, because we evaluated the patients without symptoms. Finally, this was a retrospective single‐center study with the attendant limitations.

## 6 CONCLUSION

Improvement in QOL and cardiac function after CA of asymptomatic persistent AF was revealed. According to our results, we propose that asymptomatic persistent AF should be appropriately treated by CA, although, however, our proposal should be tested prospectively.

## Disclosure statement

Authors declare no conflict of interest for this article. The study protocol was approved on May 13, 2017 by a suitably constituted Ethics Committee of Tenri Hospital (no. 429), and it conforms to the provisions of the Declaration of Helsinki.

## References

[1] Calkins H , Hindricks G , Cappato R , Kim YH , Saad EB , Aguinaga L , et al. 2017 HRS/EHRA/ECAS/APHRS/SOLAECE expert consensus statement on catheter and surgical ablation of atrial fibrillation: Executive summary. Europace. 2018;20:157–208. 29016841 10.1093/europace/eux275 PMC5892164

[2] Jaïs P , Cauchemez B , Macle L , Daoud E , Khairy P , Subbiah R , et al. Catheter ablation versus antiarrhythmic drugs for atrial fibrillation. The A4 study. Circulation. 2008;118:2498–505. 19029470 10.1161/CIRCULATIONAHA.108.772582

[3] Scherr D , Khairy P , Miyazaki S , Aurillac‐Lavignolle V , Pascale P , Wilton SB , et al. Five‐year outcome of catheter ablation of persistent atrial fibrillation using termination of atrial fibrillation as a procedural endpoint. Circ Arrhythm Electrophysiol. 2015;8:18–24. 25528745 10.1161/CIRCEP.114.001943

[4] Gage BF , Waterman AD , Shannon W , Boechler M , Rich MW , Radford MJ . Validation of clinical classification schemes for predicting stroke results from the national registry of atrial fibrillation. JAMA. 2001;285:2864–70. 11401607 10.1001/jama.285.22.2864

[5] Kirchhof P , Benussi S , Kotecha D , Ahlsson A , Atar D , Casadei B , et al. 2016 ESC Guidelines for the management of atrial fibrillation developed in collaboration with EACTS. Europace. 2016;18:1609–78. 27567465 10.1093/europace/euw295

[6] Arnar DO , Mairesse GH , Boriani G , Calkins H , Chin A , Coats A , et al. Management of asymptomatic arrhythmias: a European Heart Rhythm Association (EHRA) consensus document, endorsed by the Heart Failure Association (HFA), Heart Rhythm Society (HRS), Asia Pacific Heart Rhythm Society (APHRS), Cardiac Arrhythmia Society of Southern Africa (CASSA), and Latin America Heart Rhythm Society (LAHRS). Europace. 2019; 1–32. 30882141 10.1093/europace/euz046

[7] Yamashita T , Ogawa S , Aizawa Y , Atarashi H , Inoue H , Ohe T , et al. Investigation of the optimal treatment strategy for atrial fibrillation in Japan ‐The J‐RHYTHM (Japanese rhythm management trial for atrial fibrillation) study design. Circ J. 2003;67:738–41. 12939546 10.1253/circj.67.738

[8] Yamashita T , Kumagai K , Koretsune Y , Mitamura H , Okumura K , Ogawa S , et al. A new method for evaluating quality of life specific to patients with atrial fibrillation: Atrial fibrillation quality of life questionnaire (AFQLQ). Jpn J Electrocardiol. 2003;23:332–43 (in Japanese).

[9] Yamashita T , Komatsu T , Kumagai K , Uno K , Niwano S , Fujiki A , et al. Internal consistency and reproducibility of atrial fibrillation quality of life questionnaire (AFQLQ). Jpn J Electrocardiol. 2005;25:488–94 (in Japanese).

[10] Maeda K , Tsutamoto T , Wada A , Hisanaga T , Kinoshita M . Plasma brain natriuretic peptide as a biochemical marker of high left ventricular end‐diastolic pressure in patients with symptomatic left ventricular dysfunction. Am Heart J. 1998;135:825–32. 9588412 10.1016/s0002-8703(98)70041-9

[11] Maisel AS , Krishnaswamy P , Nowak RM , McCord J , Hollander JE , Duc P , et al. Rapid measurement of B‐type natriuretic peptide in the emergency diagnosis of heart failure. N Engl J Med. 2002;347:161–7. 12124404 10.1056/NEJMoa020233

[12] Hanazawa K , Kaitani K , Hayama Y , Onishi N , Tamaki Y , Miyake M , et al. Effect of radiofrequency catheter ablation of persistent atrial fibrillation on the left atrial function: assessment by 320‐row multislice computed tomography. Int J Cardiol. 2015;179:449–54. 25465306 10.1016/j.ijcard.2014.11.103

[13] Reant P , Lafitte S , Jaïs P , Serri K , Weerasooriya R , Hocini M , et al. Reverse remodeling of the left cardiac chambers after catheter ablation after 1 year in a series of patients with isolated atrial fibrillation. Circulation. 2005;112:2896–903. 16260634 10.1161/CIRCULATIONAHA.104.523928

[14] Lavergne F , Morin L , Armitstead J , Benjafield A , Richards G , Woehrle H . Atrial fibrillation and sleep‐disordered breathing. J Thorac Dis. 2015;7:575–84. 10.3978/j.issn.2072-1439.2015.12.57 PMC4703675 26793367

[15] Kaitani K , Kondo H , Hanazawa K , Onishi N , Hayama Y , Tsujimura A , et al. Relationship between diastolic ventricular dysfunction and subclinical sleep‐disordered breathing in atrial fibrillation ablation candidates. Heart Vessels. 2016;31:1140–7. 26129869 10.1007/s00380-015-0705-x

[16] Kumagai K , Muraoka S , Mitsutake C , Takashima H , Nakajima H . A new approach for complete isolation of the posterior left atrium including pulmonary veins for atrial fibrillation. J Cardiovasc Electrophysiol. 2007;18:1047–52. 17655662 10.1111/j.1540-8167.2007.00911.x

[17] Momomura S , Akashiba T , Asanoi H , Ando S , Kario K , Shiomi T , et al. Guidelines for diagnosis and treatment of sleep disordered breathing in cardiovascular disease (JCS 2010). Circ J. 2010;74(Suppl. II):1–31 (in Japanese).20035085

[18] Camm AJ , Kirchhof P , Lip GY , Schotten U , Savelieva I , Ernst S , et al. Guidelines for the management of atrial fibrillation: The task force for the management of atrial fibrillation of the European society of cardiology (ESC). Eur Heart J. 2010;31:2369–429. 20802247 10.1093/eurheartj/ehq278

[19] Hsu L‐F , Jaïs P , Sanders P , Garrigue S , Hocini M , Sacher F , et al. Catheter ablation for atrial fibrillation in congestive heart failure. N Engle J Med. 2004;351:2373–83. 10.1056/NEJMoa041018 15575053

[20] Weerasooriya R , Jaïs P , Hocini M , Scavée C , MacLe L , Hsu LF , et al. Effect of catheter ablation on quality of life of patients with paroxysmal atrial fibrillation. Heart Rhythm. 2005;2(6):619–623. 15922270 10.1016/j.hrthm.2005.02.1037

[21] Blomström‐Lundqvist C , Gizurarson S , Schwieler J , Jensen SM , Bergfeldt L , Kennebäck G , et al. Effect of catheter ablation vs antiarrhythmic medication on quality of life in patients with atrial fibrillation. The CAPTAF randomized clinical trial. JAMA. 2019;321:1059–68. 30874754 10.1001/jama.2019.0335 PMC6439911

[22] Ware JE Jr , Sherbourne CD . The MOS 36‐item short‐form health survey (SF‐36). I. Conceptual framework and item selection. Med Care. 1992;30:473–83. 1593914

[23] Wokhlu A , Monahan KH , Hodge DO , Asirvatham SJ , Friedman PA , Munger TM , et al. Long‐term quality of life after ablation of atrial fibrillation the impact of recurrence, symptom relief, and placebo effect. J Am Coll Cardiol. 2010;55:2308–16. 20488300 10.1016/j.jacc.2010.01.040

[24] Miyazaki S , Kuwahara T , Takahashi A , Kobori A , Takahashi Y , Nozato T , et al. Effect of left atrial ablation on the quality of life in patients with atrial fibrillation. Circ J. 2008;72:582–7. 18362429 10.1253/circj.72.582

[25] Yagishita A , Yamauchi Y , Sato H , Yamashita S , Hirao T , Miyamoto T , et al. Improvement in the quality of life and exercise performance in relation to the plasma B‐type natriuretic peptide level after catheter ablation in patients with asymptomatic persistent atrial fibrillation. Circ J. 2017;81:444–9. 28123151 10.1253/circj.CJ-16-1123

[26] Proietti R , Birnie D , Ziegler PD , Wells GA , Verma A . Postablation atrial fibrillation burden and patient activity level: insights from the DISCERN AF study. J Am Heart Assoc. 2018;7:e010256. 30486704 10.1161/JAHA.118.010256 PMC6405544

[27] Ohmatsu S , Nakano H , Tominaga T , Terakawa Y , Murata T , Morioka S . Activation of the serotonergic system by pedaling exercise changes anterior cingulate cortex activity and improves negative emotion. Behav Brain Res. 2014;270:112–7. 24815213 10.1016/j.bbr.2014.04.017

[28] Hunter RJ , Berriman TJ , Diab I , Kamdar R , Richmond L , Baker V , et al. A randomized controlled trial of catheter ablation versus medical treatment of atrial fibrillation in heart failure (The CAMTAF Trial). Circ Arrhythm Electrophysiol. 2014;7:31–8. 24382410 10.1161/CIRCEP.113.000806

[29] Ypenburg C , van Bommel RJ , Borleffs CJW , Bleeker GB , Boersma E , Schalij MJ , et al. Long‐term prognosis after cardiac resynchronization therapy is related to the extent of left ventricular reverse remodeling at midterm follow‐Up. J Am Coll Cardiol. 2009;53:483–90. 19195605 10.1016/j.jacc.2008.10.032

[30] Yu C‐M , Bleeker GB , Fung J‐H , Schalij MJ , Zhang Q , van der Wall EE , et al. Left ventricular reverse remodeling but not clinical improvement predicts long‐term survival after cardiac resynchronization therapy. Circulation. 2005;112:1580–16. 16144994 10.1161/CIRCULATIONAHA.105.538272

[31] Kuperstein R , Goldenberg I , Moss AJ , Solomon S , Bourgoun M , Shah A , et al. Left atrial volume and the benefit of cardiac resynchronization therapy in the MADIT‐CRT trial. Circ Heart Fail. 2014;7:154–60. 24347664 10.1161/CIRCHEARTFAILURE.113.000748

[32] Krisai P , Aeschbacher S , Bossard M , Herber E , Blum S , Meyre P , et al. Change in atrial fibrillation burden over time in patients with nonpermanent atrial fibrillation. Cardiol Res Pract. 2020;2020:9583409. 32377430 10.1155/2020/9583409 PMC7183533

